# Physical frailty and the risk of peripheral vertigo: evidence from the UK Biobank cohort

**DOI:** 10.3389/fneur.2026.1910905

**Published:** 2026-08-11

**Authors:** Ziyu Zhai, Yan Lv, Peipei Li, Yuan Zhang, Ling Li, Pengfei Wang, Xiangying Suo, Junru Ding, Fanglei Ye, Yacong Bo, Le Wang, Yixu Wang

**Affiliations:** 1 Department of Otolaryngology Head and Neck Surgery, Union Hospital, Tongji Medical College, Huazhong University of Science and Technology, Wuhan, China; 2 Department of Otolaryngology Head and Neck Surgery, The First Affiliated Hospital of Zhengzhou University, Zhengzhou, China; 3 Department of Nephrology, The First Affiliated Hospital of Zhengzhou University, Zhengzhou, China; 4 School of Public Health, Zhengzhou University, Zhengzhou, China; 5 Henan Medical College of Zhengzhou University, Zhengzhou, China; 6 Department of Otolaryngology Head and Neck Surgery, People’s Hospital, Peking University, Beijing, China

**Keywords:** peripheral vertigo, physical frailty, pre-frailty, sex, UK Biobank

## Abstract

**Background:**

Peripheral vertigo is a common clinical condition that can substantially impair patients’ balance and disrupt their daily and social activities. In some patients, symptoms may persist or recur and may be accompanied by auditory dysfunction or psychological problems, thereby imposing a considerable burden on individuals, families, and healthcare systems. Physical frailty, a prevalent clinical syndrome marked by reduced physiological reserve and increased stress vulnerability, is closely linked to various age-related functional impairments. Nevertheless, the specific association between physical frailty and peripheral vertigo risk remains unclear. This study aimed to explore this relationship using large-scale population-based data from the UK Biobank (UKB).

**Methods:**

Based on UKB data, this retrospective cohort study enrolled 489,266 participants. Baseline frailty was assessed using the validated Fried phenotype encompassing five core components. Incident peripheral vertigo was defined using ICD-10 codes H810, H811, H812, and H813 based on hospital inpatient records. Multivariable Cox proportional hazards models and restricted cubic spline analyses were used to evaluate independent associations and dose–response trends, while subgroup analyses were conducted to explore sex-based heterogeneity.

**Results:**

During a median follow-up of 13.2 years, 2,802 new peripheral vertigo cases were identified. After full covariate adjustment, pre-frailty (HR = 1.36, 95% CI: 1.26–1.48) and frailty (HR = 2.05, 95% CI: 1.77–2.37) were independently associated with increased peripheral vertigo risk. A significant linear dose–response relationship was observed, with each one-point increment in frailty score increasing vertigo risk by 24% (95% CI: 19–29%). All five frailty components were independently associated with an increased risk of peripheral vertigo (all *p* < 0.001). Subgroup analyses verified a prominent sex interaction, with a stronger adverse effect of frailty in females.

**Conclusion:**

Pre-frailty and physical frailty were independently and dose-dependently associated with an increased risk of incident peripheral vertigo, with all five frailty components showing significant associations with peripheral vertigo risk. The detrimental association is significantly modified by sex and more pronounced in females. This study provides novel population-based evidence supporting the potential value of frailty assessment in identifying individuals at elevated risk of peripheral vertigo, especially among females.

## 1 Introduction

Vertigo is a common, disabling clinical symptom defined by a false perception of rotational or positional movement (1, 2). It can be classified into central vertigo and peripheral vertigo (3). The clinical manifestations of peripheral vertigo vary according to the specific underlying disorder. Some patients may experience recurrent episodes of severe rotational vertigo accompanied by auditory symptoms, such as tinnitus and hearing loss, and autonomic symptoms, including nausea and vomiting (4). Typical related diseases include benign paroxysmal positional vertigo (BPPV), vestibular neuritis (VN), and Ménière’s disease (MD) (5, 6). Epidemiological findings have shown that 15–20% of the adult population worldwide experiences vertigo, with prevalence increasing with age and exhibiting a two- to three-fold higher rate in females than in males (7). Beyond acute physical discomfort, chronic recurrent peripheral vertigo significantly compromises daily function and quality of life, elevating the risks of falls (8), mobility limitation (9, 10), depression, and social withdrawal, thereby placing a substantial burden on individuals and healthcare systems (11, 12).

Frailty is an aging-related clinical syndrome resulting from cumulative physiological decline, which manifests as reduced physiological reserve and increased vulnerability to endogenous and exogenous stressors (13, 14). As a standardized and validated assessment tool, the Fried frailty phenotype evaluates frailty status based on five core indicators: slow gait speed, unintentional weight loss, low physical activity, decreased grip strength, and self-reported fatigue (15). In the context of global population aging, frailty has become a major public health challenge affecting older adult health (16, 17). Although several studies have observed a potential link between frailty and vestibular dysfunction, existing evidence remains preliminary (18). Large-scale longitudinal studies that systematically clarify the independent relationship between physical frailty and peripheral vertigo are still lacking.

In particular, population-based longitudinal evidence confirming the independent predictive role of frailty in peripheral vertigo development is currently limited (19, 20). To fill these research gaps, the present study conducted a retrospective cohort analysis using data from the UKB. We aimed to explore the association of pre-frailty and frailty status with the subsequent risk of incident peripheral vertigo. We hypothesized that individuals with pre-frailty or frailty would have an elevated risk of developing peripheral vertigo compared with non-frail individuals. Beyond general association analysis, this study further focused on the dose–response trend and sex-specific heterogeneity of the association. Collectively, our findings provide novel epidemiological evidence supporting the integration of frailty screening into routine vestibular evaluation, which may facilitate early risk recognition and optimized clinical management of peripheral vertigo.

## 2 Methods

### 2.1 Study participants

This study utilized data from the UKB, a large population-based cohort established to explore genetic, environmental, and lifestyle determinants of human diseases across the lifespan. The UKB recruited more than 500,000 participants aged 37–73 years from 22 assessment centers across England, Wales, and Scotland between 2006 and 2010 (21). Baseline information on demographics, lifestyle behaviors, and health status was collected via standardized questionnaires. We conducted a retrospective cohort study to examine the association between baseline frailty status and the subsequent risk of peripheral vertigo. After excluding 12,672 participants with missing frailty-related data and 430 participants with peripheral vertigo at baseline, 489,266 participants were included in the final analysis.

### 2.2 Assessment of baseline frailty

Baseline physical frailty was assessed using the validated Fried frailty phenotype, a widely recognized tool for evaluating physiological vulnerability in aging populations (15). This phenotype comprises five core criteria: unintentional weight loss, self-reported exhaustion, low physical activity, slow gait speed, and weak grip strength, which were originally developed based on data from the Cardiovascular Health Study. Given the slight differences in measurement items between the UKB and the original Cardiovascular Health Study, we adopted previously published analytical strategies to align UKB variables with standard Fried criteria (15). Participants were categorized into three groups according to the number of positive frailty components: non-frail (0 criterion), pre-frail (1–2 criteria), and frail (3–5 criteria). Detailed information on each frailty component and the corresponding UKB Field IDs is provided in Supplementary Table 1.

### 2.3 Outcomes

The primary outcome of the present study was incident peripheral vertigo. Peripheral vertigo was identified through hospital inpatient records using the International Classification of Diseases, 10th Revision (ICD-10) codes H810 (MD), H811 (BPPV), H812 (VN), and H813 (other peripheral vertigo), as detailed in Supplementary Table 2. Hospital admission data were available until October 2022 for England, August 2022 for Scotland, and May 2022 for Wales. Participants were followed from baseline until the first recorded diagnosis of peripheral vertigo, death, loss to follow-up, or the end of the study period, whichever occurred first.

### 2.4 Covariates

The covariates for this study were selected based on existing literature and established knowledge regarding factors that may influence frailty and vertigo. Several key socio-demographic, lifestyle, and health-related variables were included as potential confounders in the analysis. These variables comprised age, sex (female or male), body mass index (BMI), ethnicity (white or non-white), education level (college or other), Townsend deprivation index (TDI), smoking status (never, former, or current), alcohol intake (never, former, or current), and physical activity level (high, moderate, or low).

### 2.5 Statistical analyses

Baseline participant characteristics were summarized according to frailty status (non-frail, pre-frail, and frail). Categorical variables were described as counts and percentages, and continuous variables were presented as means ± standard deviations or medians with interquartile ranges based on data distribution. Multivariable Cox proportional hazards models were used to evaluate the association between frailty status and incident peripheral vertigo, with HRs and 95% CIs calculated to quantify effect sizes. Three hierarchical models were applied: Model 1 was adjusted for age and sex; Model 2 was additionally adjusted for BMI and TDI; and Model 3 was further adjusted for ethnicity, education level, smoking status, alcohol intake, and physical activity.

We further assessed the independent effects of each frailty component on peripheral vertigo incidence, adjusting for all covariates and mutual adjustment for other frailty components. Restricted cubic spline regression was performed to examine the dose–response relationship between continuous frailty scores and vertigo risk.

All analyses were conducted in R 4.3.2. All tests were two-tailed, and a *p* < 0.05 was considered statistically significant.

### 2.6 Subgroup and sensitivity analyses

To explore potential heterogeneity in the association between physical frailty and peripheral vertigo risk, subgroup analyses were performed across multiple prespecified population factors. We assessed effect modification and statistical interactions across subgroups defined by sex (female vs. male), age (<65 vs. ≥ 65 years), ethnicity (others vs. White), education level (others vs. university), physical activity level (low vs. moderate/high), employment status (no vs. yes), smoking status (no vs. yes), alcohol intake (no vs. yes), and BMI category (<25 vs. ≥ 25 kg/m^2^). Sensitivity analyses were further conducted to verify the robustness of the primary findings, mitigate the influence of residual confounders and potential biases, and improve the credibility of the results.

## 3 Results

### 3.1 Baseline characteristics of study participants

**Figure 1:**
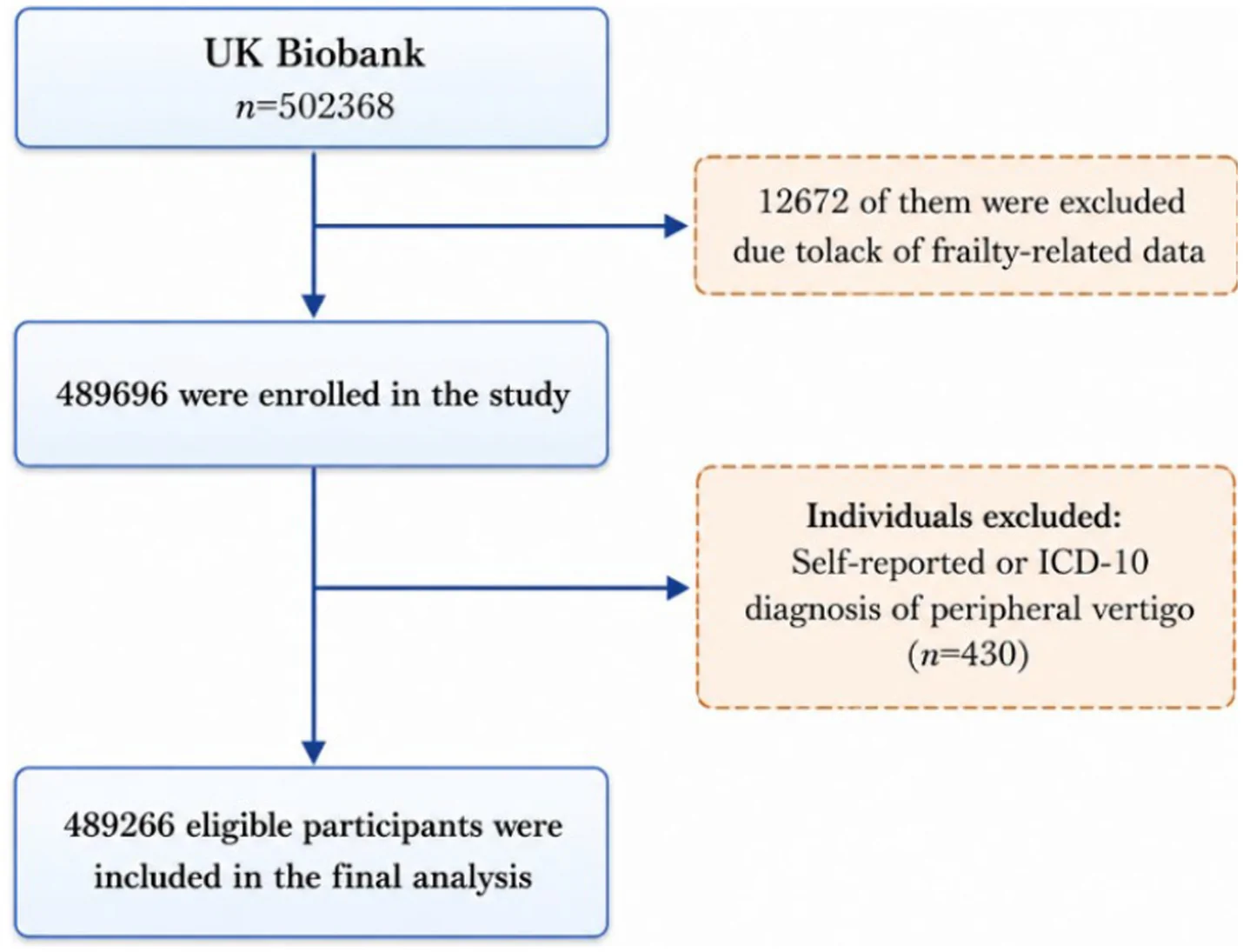
Study design and participant flow chart of the present study. A total of 502,368 participants from the UKB were initially screened. After excluding participants with missing frailty data and those with a baseline diagnosis of peripheral vertigo, 489,266 individuals were finally included in the retrospective cohort analysis. ICD, International Classification of Diseases.

A total of 502,368 participants were initially enrolled in the UKB cohort. After excluding 12,672 individuals with missing frailty-related data and an additional 430 participants diagnosed with peripheral vertigo at baseline, the final analytical population comprised 489,266 eligible participants (Figure 1). During the follow-up period covering 6,020,660 person-years, the median follow-up duration was 13.2 years, and 2,802 new cases of incident peripheral vertigo were identified across the entire cohort.

The overall mean age was 57.0 years, and females accounted for 54.4% (266,021/489,266) of the total population. The majority of participants were of White ethnicity (94.5%, 462,115/489,266). Based on the Fried frailty phenotype criteria, participants were divided into three groups: 252,193 (51.6%) non-frail individuals, 214,648 (43.9%) pre-frail individuals, and 22,425 (4.6%) frail individuals. Detailed baseline characteristics stratified by frailty status are summarized in Table 1.

**Table 1:** Characteristics of the population by the frailty phenotypes.

Characteristics	Total	Non-frailty	Pre-frailty	Frailty
Number of participants	489,266	252,193	214,648	22,425
Age, years	57.0 ± 8.1	56.5 ± 8.1	57.5 ± 8.1	58.6 ± 7.7
Sex				
Female	266,021 (54.4)	133,282 (52.8)	119,713 (55.8)	13,026 (58.1)
Male	223,245 (45.6)	118,911 (47.2)	94,935 (44.2)	9,399 (41.9)
Ethnicity				
White	462,115 (94.5)	242,814 (96.3)	199,551 (93.0)	19,750 (88.1)
Non-White	27,151 (5.5)	9,379 (3.7)	15,097 (7.0)	2,675 (11.9)
Education level				
Not college	328,717 (67.2)	158,558 (62.9)	151,546 (70.6)	18,613 (83.0)
College degree	160,549 (32.8)	93,635 (37.1)	63,102 (29.4)	3,812 (17.0)
TDI	−1.32 (3.08)	−1.71 (2.86)	−1.06 (3.18)	0.41 (3.56)
BMI, kg·m^−2^	27.42 (4.78)	26.81 (4.22)	27.84 (5.02)	30.18 (6.63)
Smoking status				
Never	268,078 (54.8)	143,372 (56.9)	114,659 (53.4)	10,047 (44.8)
Previous	169,785 (34.7)	87,203 (34.6)	74,796 (34.8)	7,786 (34.7)
Current	51,403 (10.5)	21,618 (8.6)	25,193 (11.7)	4,592 (20.5)
Alcohol intake				
Never	21,206 (4.3)	7,584 (3.0)	11,263 (5.2)	2,359 (10.5)
Previous	17,365 (3.5)	5,965 (2.4)	9,046 (4.2)	2,354 (10.5)
Current	450,695 (92.1)	238,644 (94.6)	194,339 (90.5)	17,712 (79.0)
Physical activity				
Low	91,690 (18.7)	37,430 (14.8)	45,091 (21.0)	9,169 (40.9)
Moderate	199,975 (40.9)	104,458 (41.4)	87,495 (40.8)	8,022 (35.8)
High	197,601 (40.4)	110,305 (43.7)	82,062 (38.2)	5,234 (23.3)

Data are presented as mean ± SD, *n* (%) or median (interquartile range), unless otherwise stated. TDI, Townsend deprivation index; BMI: body mass index.

Compared with non-frail participants, individuals in the pre-frail and frail groups were older, more likely to be female, and had lower educational attainment. They also presented higher Townsend deprivation index (TDI) values and higher body mass index (BMI). In terms of lifestyle factors, the prevalence of current smoking and lifelong alcohol abstinence gradually increased from the non-frail group to the frail group. Additionally, physical activity levels declined progressively across the three groups, with the frail group having the largest proportion of participants with low physical activity (40.9%).

### 3.2 Association between frailty phenotype and incident peripheral vertigo

Multivariable Cox proportional hazards regression models were applied to evaluate the independent association between frailty status and the risk of incident peripheral vertigo (Table 2). Three hierarchical adjustment models were constructed to control for potential confounders. In Model 1 (adjusted for age and sex alone), pre-frailty was associated with a 46% higher risk of peripheral vertigo (HR = 1.46, 95% CI: 1.35–1.58, *p* < 0.001), while frailty was linked to a 159% elevated risk (HR = 2.59, 95% CI: 2.26–2.97, *p* < 0.001). After further adjusting for BMI and TDI in Model 2, the associations remained statistically significant. In the fully adjusted Model 3 (additionally adjusted for ethnicity, education level, smoking status, alcohol intake and physical activity), the elevated risk persisted. Specifically, pre-frail participants had a 36% increased risk of developing peripheral vertigo (HR = 1.36, 95% CI: 1.26–1.48, *p* < 0.001), and frail participants exhibited a 105% higher risk relative to non-frail counterparts (HR = 2.05, 95% CI: 1.77–2.37, *p* < 0.001).

**Table 2:** Associations between frailty and the risk of incident peripheral vertigo.

Frailty status	Cases/person-years	Model 1^a^	Model 2^b^			Model 3^c^	
		HR (95% CI)	*p* value	HR (95% CI)	*p* value	HR (95% CI)	*p* value
Non-frailty	1,113/3,383,879	-	-	-	-	-	-
Pre-frailty	1,432/2,819,661	1.46(1.35–1.58)	< 0.001	1.38(1.28–1.5)	< 0.001	1.36(1.26–1.48)	< 0.001
Frailty	257/277,764	2.59(2.26–2.97)	< 0.001	2.18(1.89–2.51)	< 0.001	2.05(1.77–2.37)	< 0.001

^a^Adjusted for age, sex; ^b^Adjusted for model 1, body mass index and townsend deprivation index; ^c^Adjusted for model 2, ethnicity, smoking status, alcohol intake, physical activity and education level.

### 3.3 Dose–response relationship between frailty score and peripheral vertigo risk

**Figure 2:**
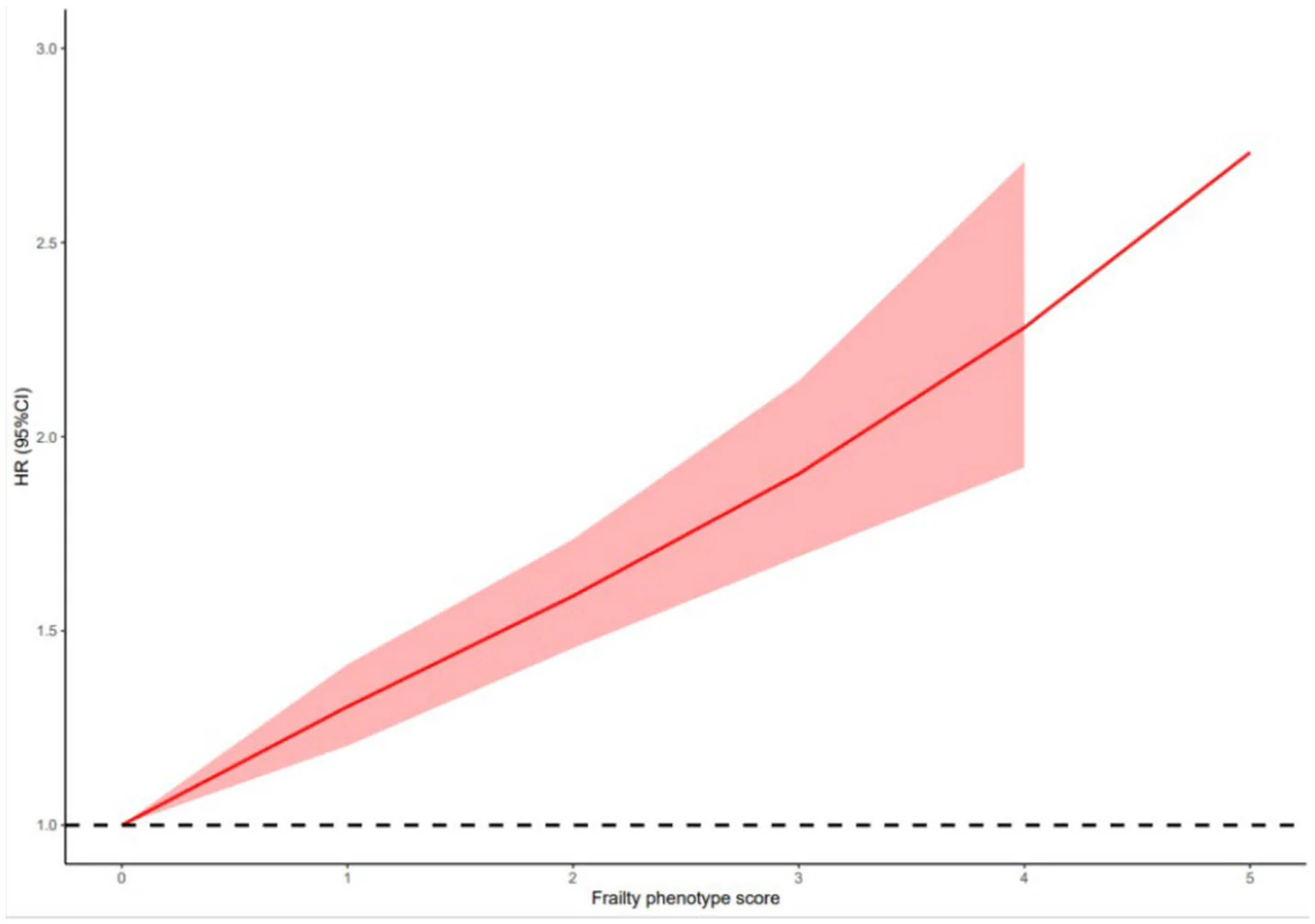
Restricted cubic spline curves illustrating the dose–response relationship between frailty phenotype score and the risk of incident peripheral vertigo. The solid line represents adjusted hazard ratios, and the shaded area indicates the 95% confidence interval. Models were fully adjusted for age, sex, ethnicity, education level, Townsend Deprivation Index, smoking status, alcohol intake, physical activity, and body mass index.

Restricted cubic spline regression was performed to explore the continuous dose–response relationship between the frailty phenotype score and the risk of incident peripheral vertigo (Figure 2). A significant linear positive correlation was observed between frailty score and vertigo risk (all *p* < 0.001). Each one-point increment in the continuous frailty score corresponded to a 24% increase in the risk of peripheral vertigo (95% CI: 19–29%). This linear trend indicated that the risk of peripheral vertigo rose steadily as the severity of physical frailty increased.

### 3.4 Associations between individual frailty components and peripheral vertigo

We further analyzed the independent effects of the five core components of the Fried frailty phenotype on peripheral vertigo incidence, with full adjustment for all predefined covariates (Table 3). All five frailty components were independently associated with a significantly higher risk of peripheral vertigo (all *p* < 0.001). Among these components, self-reported exhaustion exerted the strongest effect (HR = 1.51, 95% CI: 1.37–1.67). The remaining four components also demonstrated robust associations: slow gait speed (HR = 1.37, 95% CI: 1.22–1.54), low grip strength (HR = 1.29, 95% CI: 1.19–1.40), low physical activity (HR = 1.24, 95% CI: 1.12–1.38), and unintentional weight loss (HR = 1.21, 95% CI: 1.09–1.33).

**Table 3:** Associations between the frailty components and peripheral vertigo.

Frailty component	Cases/person-years	HR (95% CI)	*p* value
Weight loss	494/969,810	1.21 (1.09–1.33)	< 0.001
Exhaustion	492/781,814	1.51 (1.37–1.67)	< 0.001
Low physical activity	500/844,721	1.24 (1.12–1.38)	< 0.001
Slow gait speed	379/489,823	1.37 (1.22–1.54)	< 0.001
Low grip strength	832/1,334,996	1.29 (1.19–1.4)	< 0.001

Associations with a *p*-value < 0.01 were deemed statistically significant following Bonferroni correction for multiple comparisons. Adjusted for age, sex, education, body mass index, ethnicity, townsend deprivation index, smoking status, alcohol intake and physical activity.

### 3.5 Subgroup analysis and sensitivity analysis

**Figure 3:**
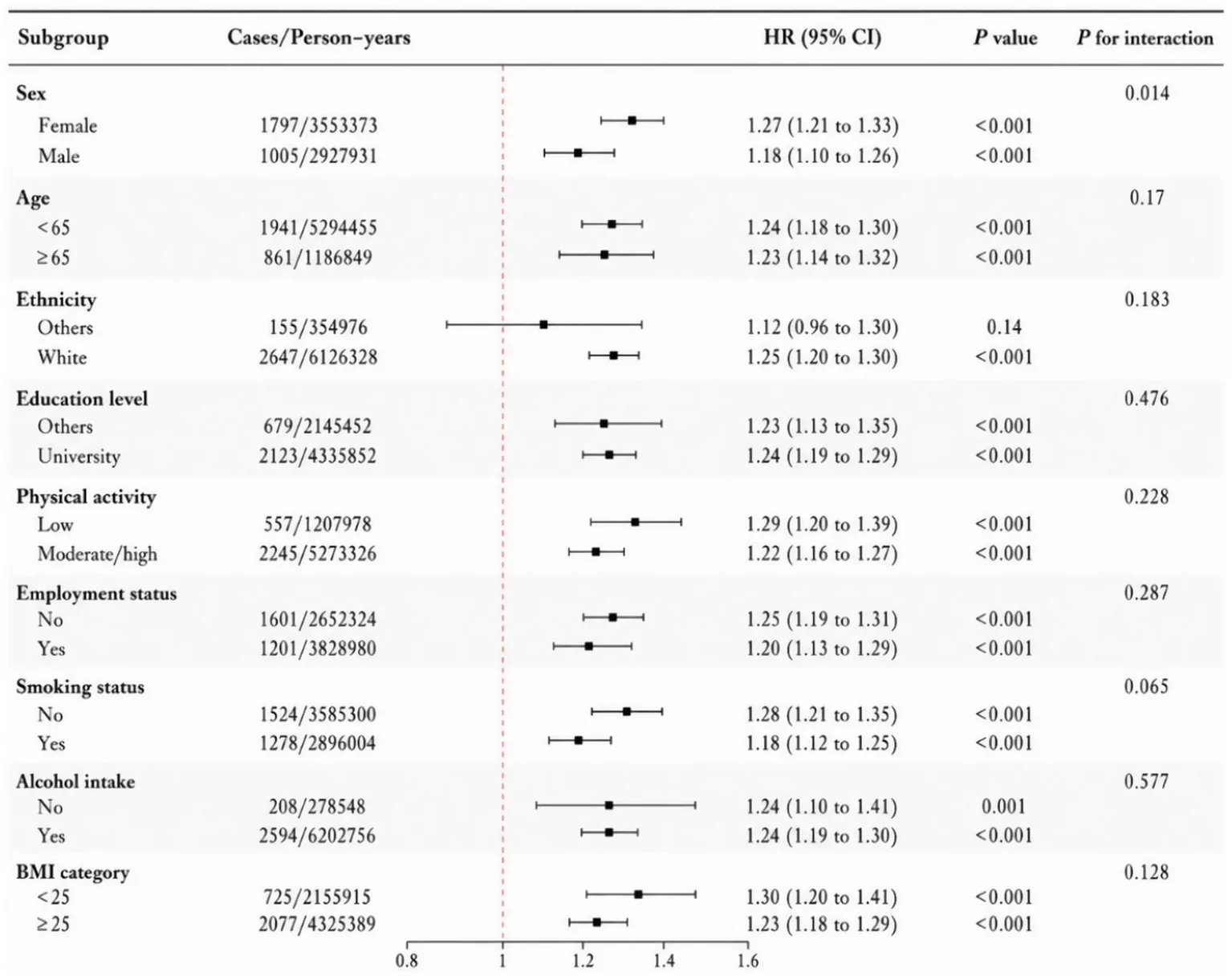
Subgroup analyses for the association between frailty and incident peripheral vertigo across prespecified stratification factors. All models were adjusted for multiple confounders. *P-int* indicates the *p* value for interaction between frailty and each subgroup factor. *HR*, hazard ratio; *CI*, confidence interval.

Stratified subgroup analyses were conducted across multiple demographic and lifestyle factors to assess potential effect modification (Figure 3). A significant sex interaction was detected (*p* for interaction < 0.05), demonstrating that the adverse association between frailty and peripheral vertigo risk was substantially stronger in female participants than in male participants.

Multiple sensitivity analyses were subsequently conducted to verify the robustness of the primary findings (Supplementary Tables 3–5). The results remained consistent across all sensitivity tests, confirming that the observed association between physical frailty and incident peripheral vertigo was reliable and not driven by residual confounding or methodological bias.

## 4 Discussion

To the best of our knowledge, this large-scale population-based cohort study is the first to systematically elaborate the independent predictive value of frailty phenotypes, dose–response trends, and sex-specific heterogeneity for incident peripheral vertigo. The results demonstrated that pre-frailty and frailty were independently associated with an increased risk of peripheral vertigo. Specifically, compared with non-frail participants, pre-frail individuals had a 36% higher risk of developing peripheral vertigo, while frail individuals exhibited a 105% increased risk. Notably, a significant linear dose–response relationship was observed, whereby increased frailty severity was progressively associated with elevated vertigo risk. Further subgroup analysis identified a prominent sex interaction, revealing that the adverse effect of frailty on peripheral vertigo risk was substantially stronger in females.

### 4.1 Frailty and risk of peripheral vertigo: an association analysis

The present study showed that both pre-frailty and frailty were independently associated with an increased risk of incident peripheral vertigo. To date, no population-based studies have systematically validated the direct association between the five core components of frailty (exhaustion, unintentional weight loss, low physical activity, slow gait speed, and reduced grip strength) and peripheral vertigo. Nevertheless, existing clinical evidence provides indirect support for our core findings. Fatigue, one of the core phenotypic features of frailty, has been reported in patients with selected peripheral vestibular disorders (22–24). A previous study enrolling 391 older adults reported dizziness in 45% of participants, among which 71.6% were female, and multivariate regression analysis verified an independent association between self-perceived fatigue and dizziness (25). In addition to fatigue, low physical activity, another key frailty component, is closely linked to peripheral vestibular disorders (26, 27). Accumulated evidence has shown that patients with BPPV generally have lower daily activity levels. It has been hypothesized that prolonged sedentary behavior and insufficient physical activity, which are prevalent in elderly populations, may accelerate otoconial degeneration, impair the stability of the otolith system, and ultimately facilitate the onset and recurrence of peripheral vertigo. However, this proposed pathway has not been directly demonstrated and therefore remains hypothetical (20). Moreover, recent studies have reported associations of BPPV with low muscle mass, reduced muscle strength, and sarcopenia-related phenotypes, providing further indirect clinical support for a link between physical frailty and selected peripheral vestibular disorders (28–30). In line with these observations, age-related vestibular decline and BPPV have also been associated with reduced physical activity, impaired gait, and an increased risk of falls, collectively suggesting a close relationship between vestibular dysfunction and physical functional decline (31–33).

Collectively, these findings provide indirect support for the association between frailty and peripheral vertigo. Future interventional studies are needed to determine whether improving frailty status can reduce the subsequent risk of peripheral vertigo.

### 4.2 Frailty and risk of peripheral vertigo: sex-specific interaction

A major novelty of this study is the identification of a significant sex interaction effect, demonstrating that the detrimental impact of frailty on peripheral vertigo risk is markedly stronger in females. Consistent with our results, previous epidemiological studies have confirmed that females have a higher prevalence of both frailty and peripheral vertigo than males (15, 34). The observed sex disparity may be partly attributable to inherent differences in physiological characteristics and psychological susceptibility between females and males.

Female-specific hormonal fluctuations, including dynamic changes in estrogen levels and menstrual cycle variation, may increase individual vulnerability to vestibular dysfunction (35, 36). In addition, females are more susceptible to affective disorders such as anxiety and depression, which have been well recognized as critical risk factors for peripheral vestibular diseases (11, 37, 38). The prevalence of depression in females is approximately twice that in males, with fluctuating incidence across the reproductive period largely driven by cyclic hormonal changes (39). Growing evidence has confirmed a close interactive relationship between emotional disorders and peripheral vertigo (40). Neuroanatomical and neurochemical studies have further revealed that the vestibular system shares overlapping brain regions and neurotransmitter regulatory pathways with emotional processing circuits, providing a potential biological mechanism explaining how emotional dysregulation aggravates vestibular dysfunction (41). Moreover, sex differences in the prevalence of specific peripheral vestibular disorders and healthcare-seeking behavior may also have contributed to the observed sex-specific association (42, 43).

Overall, the stronger association observed in females may reflect the combined influence of biological, psychological, clinical, and healthcare-related factors. These findings highlight the importance of considering sex differences when identifying individuals at increased risk of peripheral vertigo.

### 4.3 Strengths and limitations

This study has several notable strengths. To our knowledge, this is the first large-scale longitudinal cohort study to investigate the associations of pre-frailty and frailty with incident peripheral vertigo using UKB data. The large sample size and long-term prospective follow-up substantially enhance the statistical power and reliability of our results. Moreover, multiple subgroup and sensitivity analyses further supported the robustness of the observed association. Nevertheless, several limitations should be acknowledged. First, we did not perform separate analyses for different subtypes of peripheral vestibular disorders. Second, because cases were identified from hospital records, outpatient cases may have been missed; therefore, the reported incidence may not fully reflect the occurrence of peripheral vertigo across all healthcare settings. Third, residual or unmeasured confounding factors may still exist despite comprehensive covariate adjustment. Future studies with more detailed clinical phenotyping, particularly prospective interventional studies, are warranted to validate and extend the present findings and to determine whether improving modifiable frailty components can reduce the subsequent risk of peripheral vertigo and related adverse outcomes.

## 5 Conclusion

In conclusion, the present study provides novel population-based evidence that pre-frailty and frailty are independently associated with an increased risk of incident peripheral vertigo in a dose-dependent manner. The adverse association was significantly stronger in female individuals, indicating the existence of sex-specific vulnerability to vestibular dysfunction. These findings emphasize the clinical necessity of incorporating routine frailty assessment into the evaluation of vestibular disorders, particularly for older adults and female populations. Early identification of frailty may help identify individuals at elevated risk of peripheral vertigo and inform strategies to improve long-term functional outcomes and quality of life. Prospective interventional studies are warranted to further determine whether targeted frailty management can reduce the subsequent risk of peripheral vertigo.

## Data Availability

The data analyzed in this study is subject to the following licenses/restrictions: UK Biobank raw individual data is restricted by formal access agreements. Researchers need approved applications and exclusive access IDs to obtain data. Full participant datasets cannot be published in manuscripts or supplements, and redistribution/commercial use of the data is forbidden for privacy and ethical compliance. Requests to access these datasets should be directed to UK Biobank Access Management Team, https://www.ukbiobank.ac.uk/enable-your-research/apply-for-access.
