# Correction: Physical frailty and the risk of peripheral vertigo: evidence from the UK Biobank cohort

**DOI:** 10.3389/fneur.2026.1976517

**Published:** 2026-09-08

**Authors:** Ziyu Zhai, Yan Lv, Peipei Li, Yuan Zhang, Ling Li, Pengfei Wang, Xiangying Suo, Junru Ding, Fanglei Ye, Yacong Bo, Le Wang, Yixu Wang

**Affiliations:** 1 Department of Otolaryngology Head and Neck Surgery, Union Hospital, Tongji Medical College, Huazhong University of Science and Technology, Wuhan, China; 2 Department of Otolaryngology Head and Neck Surgery, The First Affiliated Hospital of Zhengzhou University, Zhengzhou, China; 3 Department of Nephrology, The First Affiliated Hospital of Zhengzhou University, Zhengzhou, China; 4 School of Public Health, Zhengzhou University, Zhengzhou, China; 5 Henan Medical College of Zhengzhou University, Zhengzhou, China; 6 Department of Otolaryngology Head and Neck Surgery, People's Hospital, Peking University, Beijing, China

**Keywords:** peripheral vertigo, physical frailty, pre-frailty, sex, UK Biobank

The author contributions statement was erroneously published as follows:

“ZZ: Conceptualization, Data curation, Formal analysis, Investigation, Methodology, Writing – original draft, Writing – review & editing. YL: Writing – original draft, Writing – review & editing. PL: Writing – original draft, Writing – review & editing. YZ: Writing – review & editing. LL: Writing – review & editing. PW: Writing – review & editing. XS: Writing – review & editing. JD: Writing – review & editing. FY: Writing – review & editing. YB: Writing – review & editing. LW: Writing – review & editing. YW: Writing – review & editing.”

The correct author contributions statement appears below:

