# Supplementary material for: Physical frailty and the risk of peripheral vertigo: evidence from the UK Biobank cohort
Source: Front Neurol. 2026 Aug 11;17:1910905. doi: 10.3389/fneur.2026.1910905 (PMC13503151; doi:10.3389/fneur.2026.1910905)
Supplement: Supplementary file 1 [file Table_1.DOCX]

**Supplemental Material**

**Table of contents**

Supplementary Table 1. Frailty definition and cut-off points in UK Biobank.

Supplementary Table 2. ICD-10 codes used to ascertain peripheral vertigo.

Supplementary Table 3. Associations between frailty and the risk of incident peripheral vertigo after excluding incident cases during the first year of follow-up (n=486,845).

Supplementary Table 4. Associations between frailty and the risk of incident peripheral vertigo with complete covariates (n= 306,274).

Supplementary Table 5. Associations between frailty and the risk of incident peripheral vertigo among participants of European descent. (n= 347,374).

**Supplementary Table 1. Frailty definition and cut-off points in UK Biobank.**

| Individual components | Criteria | Field IDs |
| --- | --- | --- |
| Weight loss | Self-reported: “Compared with one year ago, has your weight changed?”  Response: yes, lost weight = 1; other = 0;  Do not know/Prefer not to answer = missing data. | 2306 |
| Exhaustion | Self-reported: “Over the past two weeks, how often have you felt tired or had little energy?”  Response: more than half the days or nearly every day = 1; other = 0;  Do not know/Prefer not to answer = missing data. | 2080 |
| Low physical activity | Quintiles of sex- and age-specific levels of total MET minutes per week derived from IPAQ.  The lowest 20% of total MET minutes per week = 1; other = 0;  No response/ Prefer not to answer = missing data. | 31, 21022, 22037, 22038, 22039 |
| Slow gait speed | Self-reported: “How would you describe your usual walking pace?”  Response: slow = 1; other = 0;  Do not know/Prefer not to answer = missing data. | 924 |
| Low grip strength | Measured grip strength expressed in kg by sex- and BMI- adjusted cut-off points.  Cut-off points:  Male  If BMI ≤24.0 kg/m2 & grip strength ≤29 kg;  If BMI 24.1 to 26.0 kg/m2 & grip strength ≤30 kg;  If BMI 26.1 to 28.0 kg/m2 & grip strength ≤30 kg;  If BMI >28.0 kg/m2 & grip strength ≤32 kg Female  If BMI ≤23.0 kg/m2 & grip strength ≤17 kg;  If BMI 23.1 to 26.0 kg/m2 & grip strength ≤17.3 kg;  If BMI 26.1 to 29.0 kg/m2 & grip strength ≤18 kg;  If BMI >29.0 kg/m2 & grip strength ≤21 kg;  If data on BMI or grip strength is not available = missing data. | 31, 21001, 46, 47 |

Five individual components used for definition of physical frailty phenotypes are shown.

Abbreviations: BMI, body mass index; IPAQ, International Physical Activity Questionnaire; MET, metabolic equivalent.

**Supplementary Table 2. ICD-10 codes used to ascertain peripheral vertigo.**

| Diagnosis | ICD-10 codes |
| --- | --- |
| Peripheral vertigo | H810, H811, H812, H813 |

**Supplementary Table 3. Associations between frailty and the risk of incident peripheral vertigo** **after excluding incident cases during the first year of follow-up (****n=486,845).**

| Frailty status | Events/person-years | Model 1^a^ | |
| --- | --- | --- | --- |
|  |  | HR (95% CI) | *P* value |
| Non-frailty | 1054/3,382,990 | Reference |  |
| Pre-frailty | 1356/2,818,226 | 1.37(1.26,1.49) | < 0.001 |
| Frailty | 247/277,324 | 2.12(1.82,2.46) | < 0.001 |
| P value for trend |  |  | < 0.001 |

^a^ Adjusted for age, sex, ethnicity, education level, Townsend deprivation index, smoking status, alcohol intake, physical activity and [body mass index](https://www.sciencedirect.com/topics/biochemistry-genetics-and-molecular-biology/body-mass).

**Supplementary Table 4. Associations between frailty and the risk of incident peripheral vertigo** **after excluding cancer** **n=306,274).**

| Frailty status | Events/person-years | Model 1^a^ | |
| --- | --- | --- | --- |
|  |  | HR (95% CI) | *P* value |
| Non-frailty | 626/2,319,887 | Reference |  |
| Pre-frailty | 720/1,679,051 | 1.42(1.27,1.58) | < 0.001 |
| Frailty | 99/114,492 | 2.38(1.90,2.98) | < 0.001 |
| P value for trend |  |  | < 0.001 |

^a^ Adjusted for age, sex, ethnicity, education level, Townsend deprivation index, smoking status, alcohol intake, physical activity and [body mass index](https://www.sciencedirect.com/topics/biochemistry-genetics-and-molecular-biology/body-mass).

**Supplementary Table 5. Associations between frailty and the risk of incident** **peripheral vertigo with complete covariates (n=347,374).**

| Frailty status | Events/person-years | Model 1^a^ | |
| --- | --- | --- | --- |
|  |  | HR (95% CI) | *P* value |
| Non-frailty | 900/2,520,766 | Reference |  |
| Pre-frailty | 1057/1,932,090 | 1.33(1.22,1.46) | < 0.001 |
| Frailty | 168/154,064 | 2.09(1.75,2.50) | < 0.001 |
| P value for trend |  |  | < 0.001 |

^a^ Adjusted for Adjusted for age, sex, ethnicity, education level, Townsend deprivation index, smoking status, alcohol intake, physical activity and  [body mass index](https://www.sciencedirect.com/topics/biochemistry-genetics-and-molecular-biology/body-mass).
